# Synthesis and Antimicrobial Activity of Methoxy- Substituted γ-Oxa-ε-lactones Derived from Flavanones

**DOI:** 10.3390/molecules24224151

**Published:** 2019-11-16

**Authors:** Witold Gładkowski, Monika Siepka, Tomasz Janeczko, Edyta Kostrzewa-Susłow, Jarosław Popłoński, Marcelina Mazur, Barbara Żarowska, Wojciech Łaba, Gabriela Maciejewska, Czesław Wawrzeńczyk

**Affiliations:** 1 Wrocław University of Environmental and Life Sciences, Department of Chemistry, Norwida 25, 50-375 Wrocław, Poland; monikasiepka@yahoo.com (M.S.); tomasz.janeczko@upwr.edu.pl (T.J.); edyta.kostrzewa-suslow@upwr.edu.pl (E.K.-S.); jaroslaw.poplonski@upwr.edu.pl (J.P.); marcelina.mazur@upwr.edu.pl (M.M.); czeslaw.wawrzenczyk@upwr.edu.pl (C.W.); 2 Wrocław University of Environmental and Life Sciences, Department of Biotechnology and Food Microbiology, Chełmońskiego 37/41, 51-630 Wrocław, Poland; barbara.zarowska@upwr.edu.pl (B.Ż.); wojciech.laba@upwr.edu.pl (W.Ł.); 3 Central Laboratory of the Instrumental Analysis, Wroclaw University of Technology, Wybrzeże Wyspiańskiego 27, 50-370 Wroclaw, Poland; gabriela.maciejewska@pwr.edu.pl

**Keywords:** ε-lactones, chalcones, flavanones, antibacterial activity, anifungal activity, lag-phase, optical density

## Abstract

Six γ-oxa-ε-lactones, 4-phenyl-3,4-dihydro-2*H*-1,5-benzodioxepin-2-one (**5a**) and its five derivatives with methoxy groups in different positions of A and B rings (**5b**–**f**), were synthesized from corresponding flavanones. Three of the obtained lactones (**5b**,**c**,**f**) have not been previously described in the literature. Structures of all synthesized compounds were confirmed by complete spectroscopic analysis with the assignments of signals on ^1^H and ^13^C-NMR spectra to the corresponding atoms. In most cases, lactones **5a**–**f** exerted an inhibitory effect on the growth of selected pathogenic bacteria (*Escherichia coli*, *Bacillus subtilis*, and *Staphylococcus aureus*), filamentous fungi (*Fusarium graminearum*, *Aspergillus niger*, and *Alternaria* sp.), and yeast (*Candida albicans*). The broadest spectrum of activity was observed for unsubstituted lactone **5a**, which was particularly active against filamentous fungi and yeast. Lactones with methoxy groups in the 3′ (**5c**) and 4′ (**5d**) position of B ring were more active towards bacteria whereas lactone substituted in the 7 position of the A ring (**5e**) exhibited higher antifungal activity. In most cases, the introduction of lactone function increased the activity of the compound compared to its flavonoid precursors, chalcones **3a**–**e**, and flavanones **4a**–**f**.

## 1. Introduction

Antibiotic resistance is a serious problem on a global scale, becoming the biggest challenge for global health, food security, and development today. Statistics show that 70% of two million bacterial infections in United States hospitals are caused by the strains resistant to at least one drug [1]. In Great Britain, 50% of *Staphylococcus aureus* strains are resistant to methicillin. A growing number of diseases, such as tuberculosis, pneumonia, or salmonellosis, are becoming difficult to treat as antibiotics become less effective, which leads to longer hospital stays, increasing medical costs and mortality. Another serious problem are fungal diseases, which affect over a billion people. For example, 3,000,000 cases of chronic pulmonary aspergillosis, about 700,000 cases of invasive candidiasis, over 10,000,000 cases of fungal asthma, and about 1,000,000 cases of fungal keratitis have been noticed annually. Early diagnosis allows the application of proper therapy; incorrect treatment may cause complications, including serious chronic illness or even death [2].

Therefore, compounds inhibiting the growth of microorganisms are still isolated and synthesized. One of the extensively studied compounds in this field are those with lactone ring. Many groups of natural or synthetic sesquiterpenes with an α- methylene-γ-lactone ring inhibit the growth of fungi [3,4], bacteria [5], or viruses [6]. Antimicrobial activity has been proven for bicyclic γ-lactones with an alkylsubstituted cyclohexane or cyclohexene ring, including hydroxylactones and epoxylactones obtained by biotransformation [7,8,9], synthetic δ-halo-γ-lactones [10], or products of their reductive dehalogenation [11]. Simple alkylsubstituted lactones found in food as flavor substances, namely γ-decalactone, δ-decalactone, and whisky lactone, limited the growth of pathogenic strains of *Candida albicans* and *Trichophyton rubrum* [12].

**Figure 1:**
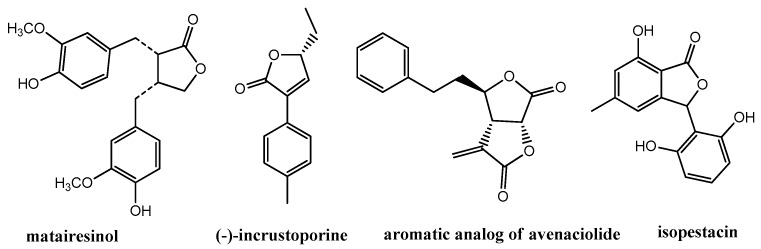
Lactones with aromatic rings exhibiting antimicrobial activity.

Antimicrobial activity is also a characteristic property of natural lactones and their synthetic analogues containing aromatic rings (Figure 1). Some lignane lactones, matairesinol and its oxidized derivatives, showed activity against some gram-negative bacteria, i.e., *Bacillus subtilis* and *Staphylococcus aureus* [13]. Antifungal activity, mainly against strains of *Candida*, was found for series of 3-(substituted phenyl)-5-alkyl-2,5-dihydrofuran-2-ones related to a natural product, (−)-incrustoporine [14,15,16]. Aromatic bis-γ-lactones analogous to avenaciolide displayed activity against *Colletotrichum gloeosporioides* [17] whereas 3,4-diphenyl-α-methylene-γ-butyrolactone turned out to be a lead scaffold for discovering compounds with high activity against *Colletotrichum lagenarium* [18]. β-Aryl-γ-lactones derived from substituted benzaldehydes inhibited the growth of some *Fusarium* strains [19] or selected bacteria [20]. An interesting example is also the strong inhibition of plant pathogenic fungi by isopestacin, the compound with the γ-lactone ring obtained from the endophytic fungus *Pestalotiopsis microspora* [21].

**Figure 2:**
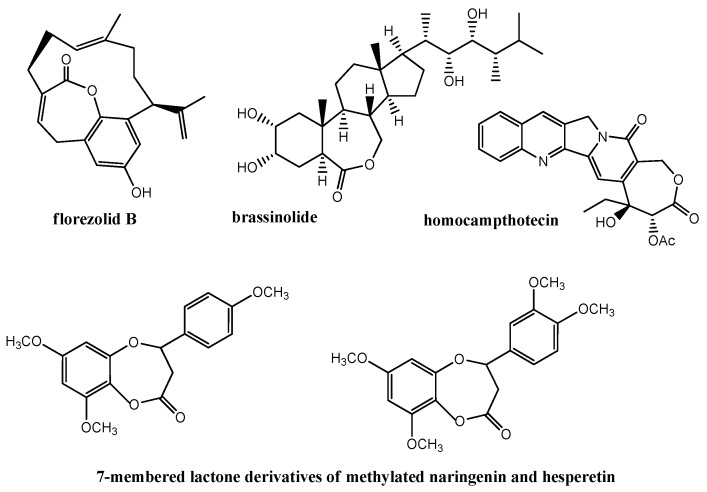
Examples of biologically active ε-lactones.

Among the biologically active lactones, only a few examples of those with a seven-membered ring are reported (Figure 2). Cytotoxic activity against KB tumor cells was proven for florezolid B isolated from an extract of an ascidian of genus *Aplidium* [22]. Numerous groups of compounds containing ε-lactone rings are brassinosteroids (e.g., brassinolide), which are essential for normal plant growth by promoting cell elongation [23]. Expanding the δ-lactone ring of natural alkaloid, camphtotecin [24], to a seven-membered lactone ring significantly increased the stability of the molecule. The obtained analogue, homocampthotecin and its derivatives, retained antiproliferative activity against different cancer cell lines [25,26].

Due to our interest in the synthesis of biologically active lactones with aromatic rings, we also paid attention to flavanone-derived ε-lactones. Some of them have been obtained earlier by the oxidation of flavanones [27,28]. Two compounds from this group, shown in Figure 2 above, were obtained from methylated naringenin and hesperetin and evaluated for apoptic activity against thee E2 human lymphoma cell line. They were found to be more active than the corresponding flavanone precursors [28].

The antimicrobial activity of chalcone **3a** and flavanone **4a** as well as their methoxy-substituted derivatives **3a**–**e** and **4a**–**f** was studied previously. Chalcones **3a**, **3b**, **3d**, and **3e** and flavanones **4a** and **4d** have been tested against methicillin-resistant *Staphylococcus aureus* [29,30], chalcone **3e** against *Candida albicans* [31], and chalcones **3e** and **3b** against *Mycobacterium tuberculosis* [32]. Flavanones **4a**, **4d**, and **4f** were tested against the following bacterial strains: *Staphylococcus aureus*, *Shiegella sonnei*, *Shiegella dysenteriae*, *Salmonella typhimurium*, *Escherichia coli*, *Vibrio cholerae*, *Vibrio parahemolyticus*, and *Enterobacter sakazakii* [33,34]; whereas flavanone **4e** was tested against the following fungal strains: *Trichoderma koningii*, *Fusarium oxysporum*, and *Cladosporium cladosporioides* [35]. In this work, we would like to present the results of the antimicrobial activity of a series of flavanone-derived γ-oxa-ε-lactones **5a**–**f** and their flavonoid precursors, the corresponding chalcones **3a**–**e** and flavanones **4a**–**f**, against selected pathogenic bacteria, filamentous fungi, and yeast. To the best of our knowledge, this kind of biological activity has not been evaluated so far for flavanone-derived ε-lactones. The main goal of this research was to determine the effect of the introduction of a lactone moiety into the flavanoid skeleton on the activity of the studied compounds.

## 2. Results and Discussion

### 2.1. Synthesis

**Scheme 1:**
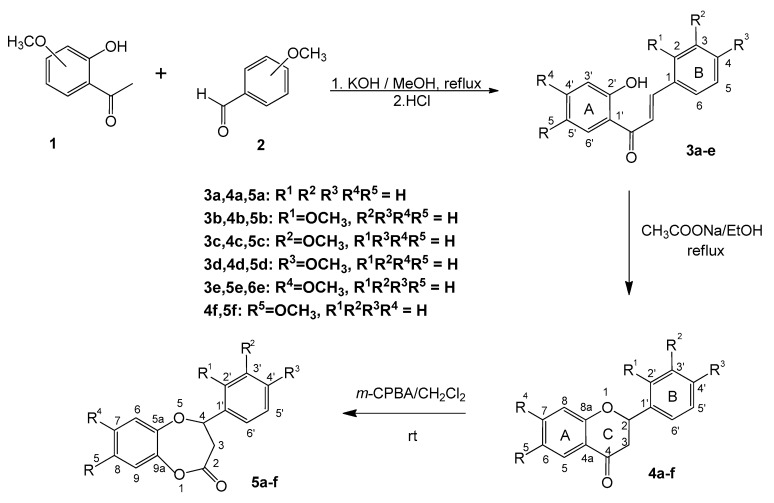
Synthesis of ε-lactones **5a**–**f.**

The γ-oxa-ε-lactones were obtained in a three-step synthesis (Scheme 1). The first step of the synthetic route was Claisen–Schmidt condensation between corresponding 2′-hydroxyacetophenones and benzaldehydes under alkaline conditions using a standard procedure [36] to afford 2′-hydroxychalcones **3a**–**e** in a 69% to 94% yield. Their physical and spectroscopic data were in accordance with detailed literature data [37,38]. Cyclization of 2′-hydroxychalcones **3a**–**e** in the presence of sodium acetate [39] yielded flavanones **4a**–**e** in a 61% to 79% yield. Synthesized flavanones **4a**–**e** and commercially available 6-methoxyflavanone **4f**, all of them characterized spectrally in the literature [37,40,41,42], were regioselectively oxidized with *m*-CPBA to corresponding γ-oxa-ε-lactones **5a**–**f** in which oxygen was introduced into the C ring between the carbonyl atom C-4 and atom C-4a.

### 2.2. Structural Analysis of ε-Lactones ***5a**–**f***

The structures of synthesized γ-oxa-ε-lactones **5a**–**f** were confirmed by spectroscopic methods (IR, NMR). Three of the synthesized lactones (**5b**,**c**,**f**) were new compounds that have not been described previously. Apart from ^1^H-NMR and ^13^C-NMR spectroscopy, we also used two-dimensional spectroscopic techniques, namely COSY, HSQC, and HMBC, to assign as many signals as possible on the NMR spectra to the corresponding protons and carbons, which in the case of spectroscopic characterization of known lactones **5a**,**d**,**e** was not reported earlier [27,43]. Full spectroscopic description of three known (**5a**,**d**,**e**) as well as three newly synthesized (**5b**,**c**,**f**) lactones was helpful for the determination of the effect of the methoxy group on the chemical shifts of corresponding atoms.

The presence of the lactone moiety was confirmed in the IR spectra by absorption bands corresponding to C=O and C-O bonds in the region of 1766 to 1773 cm^−1^ and 1205 to 1236 cm^−1^, respectively. One can see a significant difference between the absorption of carbonyl moiety in these seven-membered rings in comparison with flavanones **4a**–**f**, in which the carbonyl group absorbs in the region of 1670 to 1690 cm^−1^ [44]. On the ^1^H-NMR spectra, the protons of the CH_2_-3 methylene group are non-equivalent (diastereotopic) due to the presence of a chiral center and they couple to each other and the adjacent methine C-4 proton. As a result, their signals are observed as two doublets of doublets, with the higher (*J* = 13.2 Hz) and lower (in the range 4.8–7.3 Hz) coupling constant. These signals are located in the region of 3.00 to 3.20 ppm and the difference between their chemical shift (Δδ) is significantly lower when compared to the flavanone precursors **4a**–**f**, in which one of the protons from the methylene CH_2_-3 group is shifted upfield to the range 2.88–2.90 ppm [41]. Methine proton H-4 in lactones **5a**–**f** resonates in the region of 5.70 to 6.00 ppm and is slightly shifted upfield compared to the flavanones **4a**–**f**. Relatively small differences between vicinal coupling constants of H-4 with both methylene protons at C-3 suggest that, in these lactones, methine proton H-4 is oriented *gauche* to these protons, which implies that the phenyl substituent at C-4 is in the pseudoequatorial position. These assignments were in accordance with previous studies on flavanone-derived γ-oxa-ε-lactones [43].

Generally, in the case of lactones with unsubstituted benzene rings, most of the aromatic protons are represented by overlapping multiplets at 6.90 to 7.20 (protons from the A-ring) and 7.36 to 7.42 ppm (protons from the B-ring). However, in the spectra of two lactones with an unsubstituted A ring (**5a** and **5c**), **a** doublet from H-9 (*J* = 7.7 and 7.6 Hz, respectively) can be easily recognized because of the shielding effect of the ester group at the *ortho* position. The result of this effect is the location of H-9 signals at the higher field (7.04 and 7.07 ppm, respectively) in comparison with other aromatic protons.

Comparative analysis of spectroscopic data for methoxy-substituted derivatives **5b**–**f** showed the effect of this group on the chemical shift of the aromatic protons and carbons. As a strong electron-donating substituent, the methoxy group increases the electronic density of the benzene ring, particularly on the *ortho* and *para* positions. As a result, the protons on these positions become more shielded than the *meta*-situated protons. The observed electronic effect of the methoxy group facilitated the assignment of most of the proton signals in the NMR spectra of lactones **5b**–**f**. In all lactones **5a**–**f**, three-proton singlets from these groups are observed at 3.75 to 3.84 ppm. The location of the -OCH_3_ group at C-7 in lactone **5e** determines the chemical shifts of H-6, H-8, and H-9 protons. The signal from H-6, which is coupled only with H-8 (*J* = 2.8 Hz), is located at the higher field (6.58 ppm) because of the shielding effect of the *ortho*-situated methoxy group at C-7 and oxygen atom O-5. The signal from H-8 appears at 6.71 ppm as a doublet of doublets (*J* = 9.0 and 2.8 Hz). The doublet of H-9 is located at thee significantly lower field (7.10 ppm) as a result of the deshielding effect of the *meta*-situated methoxy group on this proton.

Substitution of the A ring with the methoxy group at C-8 (lactone **5f**) influences the chemical shifts of H-6 and H-9. The signals of these protons are visible as doublets; H-6 (*J* = 9.0 Hz) at the lower field (6.93 ppm) and H-9 (*J* = 3.0 Hz) at the higher field (6.74 ppm), which is the effect of the shielding effect of the methoxy group on the *ortho* proton H-9 and the deshielding effect of this group on the *meta* proton H-6.

The location of the methoxy group at the B ring has a significant effect on the location of signals from aromatic protons H-2′-H-5′. Taking into account the deshielding effect of this group on the protons at *meta* positions, in the spectrum of lactone **5b**, signals shifted towards the lower field, namely the triplet of doublets (*J* = 7.8 and 1.8 Hz) at 7.34 ppm and doublet of doublets (*J* = 7.8 and 1.8 Hz) at 7.61 ppm, belonging to H-4′ and H-6′, respectively. A reverse effect can be observed for protons at the *ortho* (H-3′) and *para* (H-5′) position, which are represented by a doublet of doublets (*J* = 7.8 and 0.6 Hz) at 6.93 ppm and triplet of doublets (*J* = 7.8 and 0.6 Hz) at 7.01 ppm, respectively.

In the case of lactone **5c**, the deshielding effect of the methoxy group at C-3′ applies only to proton H-5’ at the *meta* position and its signal is clearly shifted downfield (7.31 ppm), whereas other protons located at *ortho* or *para* positions are shielded by this substituent. A lack of neighboring protons allows the assignment of the singlet at 6.95 ppm to proton H-2′. Assignments of the two remaining signals to corresponding protons are possible using HMBC spectrum in which only the multiplet at 6.91 ppm correlates with signal from C-3′ which clearly shows that this multiplet comes from proton H-4′ whereas the multiplet at 6.94 ppm must be assigned to proton H-6′.

The substitution pattern of the B ring in ε-lactone **5d** generates the symmetry of this aromatic fragment of the molecule. Thus, the multiplet of protons located at the *ortho* position towards the methoxy group (H-3′, H-5′) is observed at the higher field (6.90–6.92 ppm), whereas the multiplet of protons located at *meta* positions (H-2′, H-6′) is observed at the lower field (7.28–7.29 ppm).

Many similarities can be observed in the ^13^C-NMR spectra of ε-lactones **5a**–**f**. Signals from C-2, observed at about 168 ppm, are characteristic for carbonyl atoms in seven-membered lactone rings. Signals from C-3 are located at about 38 ppm, whereas signals from C-4 are generally shifted downfield to approximately 80 ppm by the neighboring oxygen O5. In comparison with corresponding flavanones [41], in which the signal from C-4a was observed in the range 121–122 ppm, the signal of this carbon in lactones **5a**–**f** (C-9a) was significantly shifted downfield, to the range 139–146 ppm, which unequivocally confirmed the introduction of oxygen in the Baeyer–Villiger reaction between the benzene ring and the carbonyl group. Analysis of the correlations on the HSQC and HMBC spectra enabled the assignment of most of the signals at the ^13^C-NMR spectra to the corresponding carbon atoms.

### 2.3. Antimicrobial Activity Assay

Compounds were tested for their antimicrobial activity against selected strains of pathogenic bacteria (*Escherichia coli*, *Bacillus subtilis*, *Staphylococcus aureus*), filamentous fungi (*Fusarium graminearum*, *Aspergillus niger*, *Alternaria sp*.), and yeast (*Candida albicans*). The results presented in Table 1 and Table 2 show the duration of the lag phase and changes in the optical density (ΔOD), with both values determined on the basis of the resultant microbial growth curves. Two main aspects were taken into consideration analyzing the results: The effect of the location of the methoxy substituent on the activity of lactones **5a**–**f** and the comparison of the activity of lactones **5a**–**f** with their precursors: 2′-hydroxychalcones **3a**–**e** and flavanones **4a**–**f**.

#### 2.3.1. The Effect of the Methoxy Group on the Activity of Lactones **5a**–**f**

In most experiments tested, ε-lactones exerted an inhibitory effect on the growth of the tested strains by the prolongation of the lag phase and lowering the ΔOD of the microbial cultures in comparison to the controls. The broadest spectrum of activity was observed for unsubstituted compound **5a**, which completely inhibited the growth of *S. aureus* (Table 1) and all tested fungi and yeast (Table 2). The presence of the methoxy group at C-7 (lactone **5e**) did not significantly affect the mentioned activity but increased the growth inhibition of *E. coli* and *B. subtilis* (Table 1). Compared to lactone **5a**, the presence of the methoxy group in the C-8 position of the A ring (lactone **5f**) resulted in the total inhibition of *B. subtilis* (Table 1) whereas the growth of *A. niger* was not inhibited at all by this compound (Table 2). In the case of *E. coli* (Table 1) and *Alternaria sp*. (Table 2), the inhibitory effect of lactone **5f** was lower in comparison with lactone **5a**. Similar to unsubstituted ε-lactone **5a**, its derivatives with methoxy groups in the A ring (**5e**,**f**) turned out to be total growth inhibitors of the yeast *C. albicans* (Table 2). In contrast, among the three lactones with different substitution patterns of ring B, only 4′-methoxyderivative **5d** exhibited some activity against this strain. Lactones **5b**–**d** were not active against *A. niger* but inhibited strongly (**5c**) or very strongly (**5b**,**d**) the growth of *Alternaria* sp. (Table 2). In the case of *F. graminearum* (Table 2), the location of the methoxy group in the B ring affected the degree of inhibition: The most active was 2′-methoxylactone **5b** and the least active was 4′-methoxyderivative **5d**. Considering antibacterial activity (Table 1), the positive impact of the methoxy groups in the B ring was observed particularly at the 3′ and 4′ position. Complete growth inhibition of *S. aureus* and *E. coli* was found for lactones **5c** and **5d**. For lactone **5c**, a similar effect was also noticed in the case of *B. subtilis*.

#### 2.3.2. Effect of the Introduction of the Lactone Moiety into the Flavanoid Skeleton on the Antimicrobial Activity

Apart from ε-lactones, we also tested the antimicrobial activity of their corresponding precursors, 2′-hydroxychalcones **3a**–**e** and flavanones **4a**–**f**, to assess the effect of the lactone ring on the activity of flavanone-derived lactones. In many cases, ε-lactones were more active than both their precursors. Considering antibacterial activity (Table 1), the most distinct effect of the lactone ring on the inhibitory effect of the studied compounds was observed in the case of lactone **6d**, which completely inhibited the growth of *E. coli* and *S. aureus* and was a strong inhibitor of *B. subtilis*. Inhibition of these microorganisms by chalcone **3d** and flavanone **4d** was significantly lower. A positive effect of lactone function on the activity was also observed in the group of compounds with a methoxy group at position 7 of the A ring. A significantly higher inhibitory effect was noticed for 8-methoxylactone **5f** compared to its parent flavanone **4f** but only towards *B. subtilis* and *S. aureus*, whereas *E. coli* was more sensitive to flavanone **4f** than lactone **3f**. In turn, 7-methoxylactone **5e** was more active towards *S. aureus* than both chalcone **3e** and flavanone **4e**. In the case of compounds with a methoxy group at position 3′ of the B ring, both lactone **5c** and chalcone **3c** were strong inhibitors against three bacterial strains, whereas flavanone **4c** exhibited lower activity. The same dependence was observed for unsubstituted compounds **3a**,**4a**,**5a** in the tests with *S. aureus*. In some cases, ε-lactone was more active than the corresponding flavanone but less active than chalcone; in others, the following order of compounds in terms of increasing activity can be observed: Chalcone < ε-lactone < flavanone. The first case was observed for compounds with unsubstituted benzene rings **3a**,**4a**,**5a** in the tests carried out with *E. coli*; the latter case was found for the activity of compounds with the 2′-methoxy-substituted B ring (**3b**,**4b**,**5b**) towards *S. aureus* and those with a 7-methoxy-substituted A ring (**3e**,**4e**,**5e**) towards *E. coli*. Only in the case of the activity of compounds **3b**,**4b**,**5b** towards *E. coli* and *B. subtilis* and compounds **3a**,**4a**,**5a** towards *B. subtilis* did the introduction of the lactone ring not support the inhibitory effect of the compound in comparison with both chalcone and flavanone.

The most noticeable increase of antifungal activity (Table 2) after the introduction of the lactone moiety into the flavanoid molecule was observed in the case of unsubstituted compounds **4a**,**5a**,**6a**. Lactone **5a** was a significantly stronger inhibitor towards *F. graminearum* than both chalcone **3a** and flavanone **4a**; in the case of *A. niger*, *Alternaria sp*., and *C. albicans*, this activity was as strong as the activity of flavanone **4a** but still considerably higher compared with chalcone **3a**. Substitution of A ring at position 7 resulted in a significant increase of activity of lactone **5e** compared to its flavanone precursor **4e** towards filamentous fungi *A. niger*, *F. graminearum*, and *Alternaria sp*. as well as yeast *C. albicans*. In the cases of the last three strains, a comparably strong inhibition was also observed for starting chalcone **3e.** Lactone with the methoxy group at the 8 position of the A ring (**5f**) was a significantly stronger inhibitor of *F. graminearum* and *C. albicans* than flavanone **4f**, whereas a reverse relationship was found in the case of *Alternaria* sp.

The substitution pattern of the B ring also exhibited an impact on the antifungal and anti-yeast activity of lactones in relation to the corresponding flavanoid precursors. A negative effect on the activity towards filamentous fungi was found for lactones with the 3′-methoxy or 4′-methoxy substituent (**5c**,**5d**), which in most cases were weaker inhibitors than the corresponding chalcones **3c**,**3d** and flavanones **4c**,**4d.** An exception in this regard was a strong inhibition of *Alternaria sp.* by lactone **6d**, comparable with the activity of flavanone **4d** and stronger than chalcone **3d**. On the contrary, the lactone moiety of the compound with the 2′-methoxyphenyl substituent (**5b**) increased the activity towards *F. graminearum* and *Alternaria sp.* in comparison with flavanone **4b** or chalcone **3b**, respectively. Comparing the activity of compounds substituted at the B ring against *C. albicans*, one can see that 2′-methoxy-substituted lactone **5b** and 3′-methoxy-substituted lactone **5c** were not growth inhibitors in contrast to chalcone **3b** and flavanones **4b**,**c** respectively, whereas 4′-methoxy-substituted lactone **5d** decreased ΔOD to a higher extent than chalcone **3d** and flavanone **4d**.

## 3. Materials and Methods

### 3.1. Chemicals

Benzaldehyde (purity >99%) was purchased from Chempur (Piekary Śląskie, Poland). Methoxy-substituted benzaldehydes (purity 97–98%), 2′-hydroxyacetophenones (purity 97–98%), and *m*-chloroperbenzoic acid (*m*-CPBA, ≤77%) were purchased from Sigma-Aldrich (St. Louis, MO, USA), and 6-methoxyflavanone **4f** (purity 98%) was from Alfa Aesar (Kandel, Germany). Other chemicals purchased from Chempur were of analytical grade. Silica gel used for column chromatography (Kieselgel 60, 230–400 mesh) was purchased from (Merck, Darmstadt, Germany).

Hexane was purified by distillation before use for column chromatography. *m*-Chloroperbenzoic acid (*m*-CPBA) was dried immediately before use over anhydrous magnesium sulphate(VI) in the solution of methylene chloride (20 mL), then the dried solution of *m*-CPBA was filtered and concentrated under nitrogen.

### 3.2. Analysis

Analytical thin layer chromatography was carried out on silica gel-coated aluminum plates (DC-Alufolien Kieselgel 60 F_254_, Merck, Darmstadt, Germany). Spots were visualized using a solution of 1% Ce(SO_4_)_2_ and 2% H_3_[P(Mo_3_O_10_)_4_] × H_2_O in 10% H_2_SO_4_.

Gas chromatography (GC) analysis was performed on an Agilent Technologies 6890N instrument equipped with an autosampler, split injection (5:1), and Flame Ionization Detector (FID) detector using a DB-5HT column (30 m × 0.32 mm × 0.10 µm) with hydrogen as the carrier gas. For the analysis of compounds, the following temperature program was applied: Injector 200 °C, detector (FID) 280 °C, column temperature: 140 °C, 140–340 °C (rate 30 °C /min), and 340 °C (hold 1 min).

Nuclear magnetic resonance (NMR) spectra were recorded in CDCl_3_ solutions on a Bruker Avance II 600 MHz Bruker spectrometer (Bruker, Rheinstetten, Germany) with signals of residual solvent (δ_H_ = 7.26, δ_C_ = 77.0) as references for chemical shifts. Infrared spectroscopy (IR) spectra were determined using a Mattson IR 300 Thermo Nicolet spectrophotometer using KBr pellets or as neat. High-resolution mass spectra (HRMS) were recorded on a spectrometer Waters ESI-Q-TOF Premier XE (Waters Corp., Millford, MA, USA) by the electron spray ionization (ESI) technique.

The melting points (uncorrected) were determined on a Boetius apparatus.

### 3.3. General Procedure for the Synthesis of 2′-Hydroxychalcones ***3a**–**e***

A mixture of 2′-hydroxyacetophenone (1 equiv) and the corresponding benzaldehyde (1 equiv) was heated at reflux in 50 mL of 2% KOH methanolic solution and a drop of water. When the substrates reacted completely (TLC, 24–48 h), the reaction mixture was transferred to the flask containing 50 mL of water, 80 mL of 1M HCl, and 50 g of crushed ice. The resulting solution was gently mixed with a glass rod until the precipitate was formed (2–3 min). The precipitate was then filtered under reduced pressure and washed with 30 to 40 mL of water and recrystallized from ethanol. The yields of the reaction and physical and spectral data of the obtained 2′-hydroxychalcones **3a**–**e** are given below:

*2′-Hydroxychalcone* (**3a**): Obtained from 2′-hydroxyacetophenone (10.31 g, 0.075 mol) and benzaldehyde (8.03 g, 0.075 mol); yield 14.6 g (86%); yellow crystals; mp 77 °C (lit. 75–78 °C [45]) spectroscopic data in accordance with the literature [38].

*2′-Hydroxy-2-methoxychalcone* (**3b**): Obtained from 2′-hydroxyacetophenone (5.01 g, 0.037 mol) and 2-methoxybenzaldehyde (5 g, 0.037 mol); yield 7.64 g (82%); yellow crystals, mp 92–93 °C (lit. 111–112 °C [46]) spectroscopic data in accordance with the literature [38].

*2′-Hydroxy-3-methoxychalcone* (**3c**): Obtained from 2′-hydroxyacetophenone (4.02 g, 0.029 mol) and 3-methoxybenzaldehyde (4 g, 0.029 mol); yield 5.15 g (69%); yellow crystals, mp 73–75 °C (lit. 90–92 °C [46]); spectroscopic data in accordance with the literature [37].

*2′-Hydroxy-4-methoxychalcone* (**3d**): Obtained from 2′-hydroxyacetophenone (5.04 g, 0.037 mol) and 4-methoxybenzaldehyde (5 g, 0.037 mol); yield 7.83 g (84%); yellow crystals, mp 70–71 °C (lit. 70–75 °C [47]), spectroscopic data in accordance with the literature [38].

*2′-Hydroxy-4′-methoxychalcone* (**3e**): Obtained from 2′-hydroxy-4′-methoxyacetophenone (5.03 g, 0.03 mol) and benzaldehyde (3.2 g, 0.03 mol); yield 7.01 g (92%); yellow crystals, mp 93–94 °C (lit. 96–99 °C [45]); spectroscopic data in accordance with the literature [37].

### 3.4. General Procedure for the Preparation of Flavanones **4a**–**e**

A mixture of 2′-hydroxychalcone **3a**–**e** (1 equiv), sodium acetate (10 equiv), and a drop of water was heated at reflux in 200 mL of ethanol for 48 h. Then, the mixture was diluted with water and the products were extracted with methylene chloride (3 × 40 mL). The extracts were pooled, dried with anhydrous magnesium sulphate, and concentrated in vacuo. Products were crystallized from ethanol to afford pure flavanones **4a**–**e** with the following data:

*Flavanone* (**4a**): Obtained from 2′-hydroxychalcone **3a** (14.6 g, 0.065 mol); yield 11.68 g (84%); white crystals, mp 68 °C (lit. 69–72 °C [48]), spectroscopic data in accordance with the literature [40].

*2′-Methoxyflavanone* (**4b**): Obtained from 2′-hydroxy-2-methoxychalcone **3b** (7.64 g, 0.03 mol); yield 4.66 g (61%); dense oil; spectroscopic data in accordance with the literature [41].

*3′-Methoxyflavanone* (**4c**): Obtained from 2′-hydroxy-3-methoxychalcone **3c** (5.15 g, 0.02 mol); yield 3.76 g (73%); white crystals, mp 67–68 °C (lit. 78–79 °C [49]); spectroscopic data in accordance with the literature [41].

*4′-Methoxyflavanone* (**4d**): Obtained from 2′-hydroxy-4-methoxychalcone **3d** (7.83 g, 0.031 mol); yield 5.01 g (64%); white crystals, mp 74–75 °C (lit. 75–77 °C [50]), spectroscopic data in accordance with the literature [41].

*7-Methoxyflavanone* (**4e**): Obtained from 2′-hydroxy-4′-methoxychalcone **3e** (7.01 g, 0.028 mol); yield 5.47 g (78%); white crystals, mp 77–78 °C (lit. 62–63 °C [51]); spectroscopic data in accordance with the literature [37].

### 3.5. General Procedure for Preparation of γ-Oxa-ε-Lactones **5a**–**f**

Flavanone **4a**–**f** (1 equiv) and freshly dried *m*-CPBA (3 equiv) were dissolved in methylene chloride (200 mL). The mixture was stirred at room temperature until the substrate was totally consumed (48 h). The reaction was monitored by gas chromatography and TLC (hexane/acetone, 3:1). The reaction mixture was then diluted with diethyl ether, and successively washed with saturated solutions of sodium sulphite and sodium carbonate. Ethereal layer was dried over anhydrous magnesium sulphate and the solvent was evaporated in vacuo. Crystallization from hexane afforded pure lactones **5a**–**f**. Their physical and spectral data are given below:

*4-Phenyl-3,4-dihydro-2H-1,5-benzodioxepin-2-one* (**5a**): Obtained from flavanone **4a** (11.68 g, 0.05 mol); yield 9.72 g (89%); white crystals, mp 81–83 °C (lit. 85–86 °C [27]); ^1^H-NMR (600 MHz, CDCl_3_) δ: 3.11 (dd, *J* = 13.2 and 7.2 Hz, 1H, one of CH_2_-3), 3.16 (dd, *J* = 13.2 and 6.0 Hz, 1H, one of CH_2_-3), 5.73 (dd, 1H, *J* = 7.2 and 6.0 Hz, 1H, H-4), 7.04 (d, *J* = 7.7 Hz, 1H, H-9), 7.14–7.20 (m, 3H, H-6, H-7, H-8), 7.36–7.42 (m, 5H, -C_6_H_5_); ^13^C-NMR (150 MHz, CDCl_3_) δ: 38.61 (C-3), 83.59 (C-4), 120.56 (C-6), 124.39 (C-9), 125.82 (C-8), 126.28 (C-2′ and C-6′), 126.67 (C-7), 128.93 (C-3′ and C-5′), 129.17 (C-4′), 138.69 (C-1′), 145.28 (C-5a), 145.77 (C-9a), 167.59 (C-2); IR (KBr, cm^−1^): 1770, 1485, 1451, 1250, 1225, 1040, 972, 765, 711.

*4-(2′-Methoxyphenyl)-3,4-dihydro-2H-1,5-benzodioxepin-2-one* (**5b**): Obtained from 2′-methoxyflavanone **4b** (4.66 g, 0.018 mol); yield 4.37 g (90%); white crystals, mp 141–142 °C; ^1^H-NMR (600 MHz, CDCl_3_) δ: 3.01 (dd, *J* = 13.2 and 4.8 Hz, 1H, one of CH_2_-3), 3.20 (dd, *J* = 13.2 and 6.0 Hz, 1H, one of CH_2_-3), 3.84 (s, 3H, -OCH_3_), 6.02 (dd, 1H, *J* = 6.0 and 4.8 Hz, H-4), 6.93 (dd, *J* = 7.8 and 0.6 Hz, 1H, H-3′), 7.01 (td, *J* = 7.8 and 0.6 Hz, 1H, H-5′), 7.13–7.19 (m, 4H, H-6, H-7, H-8, H-9), 7.34 (td, *J* = 7.8 and 1.8 Hz, 1H, H-4′), 7.61 (dd, *J* = 7.8 and 1.8 Hz, 1H, H-6′); ^13^C-NMR (150 MHz, CDCl_3_) δ: 37.89 (C-3), 55.47 (-OCH_3_), 78.52 (C-4), 110.58 (C-3′), 120.55, 123.98, 125.58 and 126.60 (C-6, C-7, C-8, C-9), 120.84 (C-5′), 126.29 (C-6′), 127.19 (C-1′), 129.87 (C-4′), 145.76 (C-9a), 146.27 (C-5a), 155.72 (C-2′), 167.89 (C-2); IR (KBr, cm^−1^): 1772, 1493, 1438, 1272, 1205, 1037, 764. HRMS: calcd for C_16_H_14_O_4_[M + Na]^+^: 293.0790, found: 293.0792.

*4-(3′-Methoxyphenyl)-3,4-dihydro-2H-1,5-benzodioxepin-2-one* (**5c**): Obtained from 3′-methoxyflavanone **4c** (3.76 g, 0.015 mol); yield 2.68 g (67%); dense oil; ^1^H-NMR (600 MHz, CDCl_3_) δ: 3.09 (dd, *J* = 13.2 and 7.3 Hz, 1H, one of CH_2_-3), 3.15 (dd, *J* = 13.2 and 5.3 Hz, 1H, one of CH_2_-3), 3.82 (s, 3H, OCH_3_), 5.69 (dd, *J* = 7.3 and 5.3 Hz, 1H, H-4), 6.91 (m, 1H, H-4′), 6.94 (m, 1H, H-6′), 6.95 (s, 1H, H-2′), 7.07 (d, *J* = 7.6 Hz, 1H, H-9), 7.15–7.19 (m, 3H, H-6, H7, H-8), 7.31 (m, 1H, H-5′); ^13^C-NMR (150 MHz, CDCl_3_) δ: 38.66 (C-3), 55.45 (OCH_3_), 83.45 (C-4), 112.00 (C-2′), 114.55 (C-4′), 118.41 (C-6′), 120.57 and 125.84 (C-7 and C-8), 124.38 (C-9), 126.68 (C-6), 130.01 (C-5′), 140.28 (C-1′), 145.30 (C-5a), 145.73 (C-9a), 159.99 (C-3′), 167.56 (C-2); IR (KBr, cm^−1^): 1773, 1490, 1437, 1268, 1211, 1035, 765. HRMS: calcd for C_16_H_14_O_4_[M + Na]^+^: 293.0790, found: 293.0794.

*4-(4′-Methoxyphenyl)-3,4-dihydro-2H-1,5-benzodioxepin-2-one* (**5d**): Obtained from 4′-methoxyflavanone **4d** (5.01 g, 0.02 mol); yield 4.20 g (79%); white crystals; mp 108–109 °C (lit. 121–122 °C, [27]); ^1^H-NMR (600 MHz, CDCl_3_) δ: 3.09 (dd, *J* = 13.2 and 6.6 Hz, 1H, one of CH_2_-3), 3.12 (dd, *J* = 13.2 and 7.2 Hz, 1H, one of CH_2_-3), 3.82 (s, 3H, -OCH_3_), 5.69 (dd, *J* = 7.2 and 6.6 Hz, 1H, H-4), 6.90–6.92 (m, 2H, H-3′ and H-5′), 6.90–7.18 (m, 4H, H-6, H-7, H-8, H-9), 7.28–7.29 (m, 2H, H-2′ and H-6′); ^13^C-NMR (150 MHz, CDCl_3_) δ: 38.54 (C-3), 55.46 (-OCH_3_), 83.39 (C-4), 114.22 (C-3′ and C-5′), 120.46, 124.52, 125.77 and 126.60 (C-6, C-7, C-8, C-9), 127.77 (C-2′ and C-6′), 130.73 (C-1′), 145.13 (C-5a), 145.87 (C-9a), 160.21 (C-4′), 167.78 (C-2); IR (KBr, cm^−1^): 1770, 1488, 1437, 1258, 1210, 1030, 766.

*7-Methoxy-4-phenyl-3,4-dihydro-2H-1,5-benzodioxepin-2-one* (**5e**); Obtained from 7-methoxyflavanone **4e** (5.47 g, 0.022 mol); yield 4.01 g (69%); white crystals, mp 71–73 °C (lit. 72–74 °C [27]); ^1^H-NMR (600 MHz, CDCl_3_) δ: 3.09–3.15 (two m, 2H, CH_2_-3), 3.75 (s, 3H, -OCH_3_), 5.72 (m, 1H, H-4), 6.58 (d, *J* = 2.8 Hz, 1H, H-6), 6.71 (dd, *J* = 9.0 and 2.8 Hz, 1H, H-8), 7.10 (d, *J* = 9.0 Hz, 1H, H-9), 7.37–7.42 (m, 5H, H-2′, H-3′, H-4′, H-5′, H-6′); ^13^C-NMR (150 MHz, CDCl_3_) δ: 38.64 (C-3), 55.89 (-OCH_3_), 83.50 (C-4), 109.63 (C-6), 110.79 (C-8), 120.87 (C-9), 126.27 (C-2′ and C-6′), 128.96 (C-3′ and C-5′), 129.19 (C-4′), 138.82 (C-1′), 139.25 (C-9a), 145.92 (C-5a), 157.90 (C-7), 168.07 (C-2); IR (KBr, cm^−1^): 1768, 1500, 1470, 1260, 1211, 1027, 703.

*8-Methoxy-4-phenylo-3,4-dihydro-2H-1,5-benzodioxepin-2-one* (**5f**): Obtained from 6-methoxyflavanone **4f** (3.0 g, 0.012 mol); yield 2.36 g (74%); white crystals; mp 122–123 °C; ^1^H-NMR (600 MHz, CDCl_3_) δ: 3.09 (dd, *J* = 13.2 and 7.2 Hz, 1H, one of CH_2_-3), 3.15 (dd, *J* = 13.2 and 6.0 Hz, 1H, one of CH_2_-3), 3.80 (s, 3H, -OCH_3_), 5.67 (dd, *J* = 7.2 and 6.0 Hz, 1H, H-4), 6.67 (dd, *J* = 9.0 and 3.0 Hz, 1H, H-7), 6.74 (d, *J* = 3.0 Hz, 1H, H-9), 6.93 (d, *J* = 9.0 Hz, 1H, H-6), 7.37–7.39 (m, 5H, H-2′, H-3′, H-4′, H-5′, H-6′); ^13^C-NMR (150 MHz, CDCl_3_) δ: 38.60 (C-3), 55.94 (-OCH_3_), 83.70 (C-4), 105.98 (C-9), 111.63 (C-7), 124.58 (C-6), 126.36 (C-2′ and C-6′), 128.90 (C-3′ and C-5′), 129.13 (C-4′), 138.64 and 138.71 (C-1′ and C-5a), 146.27 (C-9a), 157.32 (C-8), 167.65 (C-2); IR (KBr, cm^−1^): 1766, 1502, 1468, 1445, 1260, 1236, 1028, 764, 704. HRMS: calcd for C_16_H_14_O_4_[M + Na]^+^: 293.0790, found: 293.0790.

### 3.6. Antimicrobial Activity Assay

Antimicrobial tests were carried out using the bacteria strains: *Escherichia coli* PCM 2560, *Bacillus subtilis* B5, *Staphylococcus aureus* D1; filamentous fungi: *Fusarium graminearum* 109, *Aspergillus niger* XP, *Alternaria sp.*, and yeast *Candida albicans* KL-1. All strains originated from the collection of the Department of Biotechnology and Food Microbiology, Wroclaw University of Environmental and Life Sciences.

The tests were performed on the automated Bioscreen C system (Automated Growth Curve Analysis System, Lab Systems, Finland). The working volume in the wells of the Bioscreen plate was adjusted to 200 μL, containing 180 μL of culture medium and 10 μL of cell or spore solution. The final density of the cultures was 1 × 10^6^ cells mL^−1^. The bacteria were cultivated for 48 h in a liquid medium composed of nutrient broth (Biocorp) 15 g · L^−1^ and glucose 10 g · L^−1^. Yeast and fungi were cultured in YPG medium containing 10 g · L^−1^ of yeast extract, 10 g · L^−1^ of bacteriological peptone, and 10 g · L^−1^ of glucose for 48 and 96 h, respectively. Tested compounds were added to the cultures as 0.1% solutions in 10 μL of dimethyl sulfoxide (DMSO) (*w/v*). The cultures were carried out for 2 to 3 days at 30 °C (bacteria, yeasts) or 25 °C (filamentous fungi), under constant agitation. The optical density of the cell suspensions were measured automatically at the 560-nm wavelength at 30-min intervals. Each culture was performed in three replicates. The results (Table 1 and Table 2) were analyzed using spreadsheet software (Excel 97) and the means for the triplicates of each culture type were calculated. Statistics on a completely randomized design were determined using the one-way analysis of variance (ANOVA) procedure at a level of significance set at *p* < 0.050. A comparison of average growth OD microorganisms relative to the control was carried out using Dunnett’s test.

## 4. Conclusions

It was shown that γ-oxa-ε-lactones **5a**–**f** containing methoxy groups with different substitution patterns in the A and B ring inhibited the growth of selected bacteria, filamentous fungi, and yeast. The most sensitive strains were *S. aureus*, *F. graminearum*, and *Alternaria* sp. whereas the most resistant one turned out to be *A. niger*. The broadest spectrum of activity was observed for unsubstituted lactone **5a**, which was a particularly strong inhibitor of fungi and yeast. The activity and selectivity of the methoxy-substituted lactones depended on the substitution pattern of the A and B rings. A-ring substitution with a methoxy group in position 7 improved the activity and selectivity against fungal strains and yeast whereas substitution at position 8 increased the antibacterial activity. Lactones possessing methoxy groups in the 3′ and 4′ position of the B ring were highly active against bacteria whereas the compound with the methoxy group at the 2′ position was a strong inhibitor of *F. graminearum* and *Alternaria sp*. In most cases, the activity of the compounds was improved after introduction of the lactone function into the molecule compared to their flavonoid precursors, chalcones **3a**–**e** and/or flavanones **4a**–**f**. The most spectacular example was 4′-methoxy-substituted lactone **5d**, which exhibited significantly higher activity against all bacterial strains than both its flavonoid precursors. Unsubstituted lactone **5a** was a much stronger inhibitor of filamentous fungi and yeast than the starting chalcone **3a** whereas the activity of 7-methoxy lactone **5e** was significantly higher than flavanone **4e** towards these microorganisms. The γ-Oxa-ε-lactone scaffold derived from the flavanone skeleton is a good candidate for the screening of compounds with high antimicrobial activity. Our results indicate that future studies on the antifungal flavanone-derived lactones should focus on compounds substituted in the A ring whereas a candidate for an antibacterial drug will probably be a derivative substituted in the B ring.

## Figures and Tables

**Table 1:** Effect of chalcones **3a**–**e**, flavanones **4a**–**f**, and ε-lactones **5a**–**f** on the growth of selected bacterial strains.

Compound		*Escherichia coli*		*Bacillus subtilis*		*Staphylococcus aureus*	
		Lag-Phase [h]	ΔOD ^1^	Lag-Phase [h]	ΔOD	Lag-Phase [h]	ΔOD
chalcones	**3a**	n.a.^3^	0 ± 0.01 ^2^	13.5	0.15 ± 0.02	n.a.	0 ± 0.01
	**3b**	29	0.34 ± 0.09	n.a.	0 ± 0.01	4.5	0.36 ± 0.04
	**3c**	n.a.	0 ± 0.03	n.a.	0 ± 0.01	n.a.	0 ± 0.03
	**3d**	10	0.31 ± 0.08	15	0.79 ± 0.20	17.5	0.32 ± 0.06
	**3e**	14.5	0.52 ± 0.09	n.a.	0 ± 0.03	21	0.31 ± 0.07
flavanones	**4a**	4.5	0.42 ± 0.05	5.5	0.76 ± 0.09	11	0.86 ± 0.03
	**4b**	22.5	0.26 ± 0.06	31.5	0.35 ± 0.08	33.5	0.31 ± 0.06
	**4c**	21	0.44 ± 0.01	31.5	0.60 ± 0.04	30.5	0.63 ± 0.03
	**4d**	28	0.27 ± 0.01	15	0.78 ± 0.09	29.5	0.26 ± 0.06
	**4e**	27	0.26 ± 0.06	n.a.	0 ± 0.07	5	0.50 ± 0.09
	**4f**	30	0.30 ± 0.07	13	0.57 ± 0.09	22.5	0.27 ± 0.02
ε-lactones	**5a**	21	0.43 ± 0.05	7	1.04 ± 0.03	n.a.	0 ± 0.01
	**5b**	12	0.72 ± 0.08	21	0.44 ± 0.04	21	0.38 ± 0.06
	**5c**	n.a.	0 ± 0.07	n.a.	0 ± 0.02	n.a.	0 ± 0.05
	**5d**	n.a.	0 ± 0.09	17.5	0.31 ± 0.09	n.a.	0 ± 0.04
	**5e**	22	0.33 ± 0.08	7.5	0.77 ± 0.09	28	0.25 ± 0.06
**5f**		13.5	0.58 ± 0.05	n.a.	0 ± 0.04	n.a	0 ± 0.03
Control cultures ^4^		2	1.77 ± 0.09	3	1.27 ± 0.10	4	1.58 ± 0.11

^1^ Results presented as means ± standard deviation (SD). ^2^ Underlined number means the average significantly different from control. ^3^ n.a.—not applicable. ^4^ Control cultures were cultivated in the medium supplemented with DMSO without tested compounds

**Table 2:** Effect of chalcones **3a**–**e**, flavanones **4a**–**f**, and ε-lactones **5a**–**f** on the growth of selected filamentous fungi and yeast.

Compound		*Fusarium graminearum*		*Aspergillus niger*		*Alternaria sp.*		*Candida albicans*	
		Lag-Phase [h]	ΔOD ^1^	Lag-Phase [h]	ΔOD	Lag-Phase [h]	ΔOD	Lag-Phase [h]	ΔOD
**chalcones**	**3a**	26	1.45 ±0.01 ^2^	15	1.69 ± 0.01	42	0.28 ± 0.06	39	0.26 ± 0.09
	**3b**	n.a.^3^	0 ± 0.04	15	1.36 ± 0.12	48	0.21 ± 0.07	n.a.	0 ± 0.03
	**3c**	46	0.51 ± 0.09	24.5	0.61 ± 0.07	n.a.	0 ± 0.01	14	1.12 ± 01
	**3d**	58.5	0.23 ± 0.06	17.5	1.07 ± 0.09	57	0.12 ± 0.03	20.5	0.88 ± 0.10
	**3e**	n.a.	0 ± 0.07	14	0.68 ± 0.06	n.a.	0 ± 0.01	n.a.	0 ± 0.04
**flavanones**	**4a**	22	0.36 ± 0.09	n.a.	0 ± 0.06	n.a.	0 ± 0.07	n.a.	0 ± 0.05
	**4b**	42	0.12 ± 0.02	17.5	0.67 ± 0.09	n.a.	0 ± 0.06	18	0.33 ± 0.06
	**4c**	28	0.16 ± 0.08	31.5	0.35 ± 0.08	n.a.	0 ± 0.01	16	0.53 ± 0.08
	**4d**	n.a.	0 ± 0.01	27	0.38 ± 0.04	n.a.	0 ± 0.01	23	0.49 ± 0.07
	**4e**	53	0.18 ± 0.06	13	1.51 ± 0.09	9.5	0.32 ± 0.01	13	1.66 ± 0.06
	**4f**	27.5	0.60 ± 0.02	36	1.36 ± 0.10	n.a.	0 ± 0.01	15.5	0.52 ± 0.09
**ε-lactones**	**5a**	n.a.	0 ± 0.01	n.a.	0 ± 0.01	n.a.	0 ± 0.01	n.a.	0 ± 0.04
	**5b**	n.a.	0 ± 0.01	15	1.48 ± 0.02	n.a.	0 ± 0.06	16.5	1.16 ± 0.07
	**5c**	35	0.17 ± 0.01	20	0.85 ± 0.02	46	0.58 ± 0.07	20	1.22 ± 0.08
	**5d**	46.5	0.43 ± 0.08	13	2.13 ± 0.10	n.a.	0 ± 0.01	10	0.22 ± 0.06
	**5e**	n.a.	0 ± 0.03	43	0.20 ± 0.02	n.a.	0 ± 0.01	n.a.	0 ± 0.04
	**5f**	n.a.	0 ± 0.00	15	1.34 ± 0.02	28	0.70 ± 0.05	n.a.	0 ± 0.05
Control cultures ^4^		12.5	0.81 ± 0.01	12.5	1.19 ± 0.17	9	1.04 ± 0.09	8	1.03 ± 0.01

^1^ Results presented as means ± Standard Deviation (SD). ^2^ Underlined number means the average significantly different from control. ^3^ n.a.—not applicable. ^4^ Control cultures were cultivated in the medium supplemented with DMSO without tested compounds
